# Droughts, fires, and floods: the Americas need help

**DOI:** 10.1016/j.lana.2021.100097

**Published:** 2021-10-07

**Authors:** The Lancet Regional Health - Americas

Despite the ongoing COVID-19 pandemic and the fact that the world´s eyes and ears were almost entirely focused on its outcomes, the climate change crisis has never ceased to exist, with troublesome consequences. Natural and social disasters have been occurring with more intensity. In the past few months, the Americas have suffered from major climate events, such as the Hurricane Ida in the USA and major drought in Brazil. As the temperatures rise, the world is on the edge of an irreversible situation, and actions from governments are called for.

Brazil is currently suffering from the worst drought in over 100 years. Additionally, many conserved areas throughout the country such as the Amazon forest and the Pantanal wetlands are burning to the ground mostly due to anthropogenic activities. With an increase in global temperatures, fires worsen, with a direct impact on pollution and rain production. To ameliorate the situation, some of the Brazilian Government's responses to extinguish the fires in August, 2021, consisted of training army men, investing in helicopters and planes, and forbidding the use of fires in the areas for 120 days. These reactive measures were inadequate as more than 700 000 hectares were already consumed by the fire in the Pantanal area alone since January 2021. In 2020, more than 20,000 animals died in fires, and the number is still unknown for 2021. Now, as summer approaches in the southern hemisphere, the consequences of inaction kick in: Brazil, one of the richest countries in water resources, is in a critical water crisis. The expected rains did not occur this year, which have a direct impact, not only on water and energy supply, but also for all the biomes throughout the country.

Other climate crises are occurring with more frequency and intensity throughout the Americas. The 4-year US withdrawal from the global climate community has taken its toll. The USA is the second largest greenhouse gas emitter and responsible for producing more than 5 billion metric tons of carbon dioxide per year since 1990. Although the hurricane season is expected yearly, there was a rapid development of Hurricane Ida in August 2021. This was not just a random occasion. Warmer-than-usual temperatures from the Gulf of Mexico, due to ocean-level increases, sped up the process, leading to a class-4 hurricane. Hurricane Ida first hit the state of Louisiana, resulting in major floods, power outages and destruction, and a death toll of 26 people, 16 years after Hurricane Katrina´s destruction in New Orleans and just one year after Hurricane Laura. Although it lost its force, Hurricane Ida did not stop in Louisiana; because of warmer temperatures in the atmosphere, the aftermath of this climate event resulted in major floods in four other US states: New York, New Jersey, Pennsylvania, and Connecticut. At the time of writing, 50 people were confirmed dead in these four states. The occurrence of slower and stronger hurricanes can be progressively frequent as the climate crisis worsens.

Although we mainly relate climate urgency to extreme weather events, the direct impact on our health is extensive. With major episodes of wildfires and drought, respiratory diseases are prone to be abundant due to toxic air and the exposure of pollutants. The produced smoke is full of particulate matter from the wildfire, which can be associated with respiratory morbidity, such as asthma, chronic obstructive pulmonary disease, bronchitis, and pneumonia. As wildfires occur more frequently, the incidence of respiratory diseases will be higher every year and those who live close to the area will be most affected. Additionally, with the rise of water levels and an increase in global temperatures, flooding will be increasingly frequent, especially in coastal areas. The occurrence of waterborne infectious diseases, stress, malnutrition, and lack of access to health care will become more frequent, hitting especially those who are most vulnerable, such as children and older people. Although concerning, infections are not the only issue. Fires and floods lead not only to the loss of homes, belongings, and jobs of many, but also of many family and friends. The impact on mental health is considerable, leading to an increase in depression and anxiety, for example. In the areas where houses were destroyed, impoverishment will be prone to build up, thus escalating homelessness and violence. As these climate events occur, people will become more vulnerable and inequalities will be even more evident. Mortality rates will increase yearly if no serious actions are taken.

The impact of climate crises is being felt throughout the Americas and immediate responses are required. The greenhouse emissions are higher than ever in Brazil due to the increase in wildfires, deforestation, and unsustainable agriculture. Urgent actions such as forest conservation, renewable energy supply, and sustainable and efficient agriculture methods are needed. The US climate status is not much better than Brazil. After leaving the Paris Agreement for 4 years, the USA is now facing many challenges and will need to step up. One of the President´s target is to cut down greenhouse emissions by 50–52% by 2030. However, promises in themselves are not enough: each country should be held accountable for the outcomes of their climate responses, as the examples cited above are consequences of years of negligence.

Regardless of many warnings from climate research scientists and the wider communities, the whole world is still suffering from the general lack of immediate actions to address the climate crisis. The next UN Climate Change Conference – COP26 in October, 2021, will see how much and how far governments around the world are willing to commit to global climate targets and individual countries’ actions. For low-income and middle-income countries, the situation is even more critical with financial resources lacking amid the COVID-19 pandemic, and the challenges for tackling climate measures are even greater. The Americas are home to diverse human populations, and stark disparities in the access to resources exist between countries in the region and even within a single country. Climate challenges can serve as a catalyst for every country in the Americas to unite and help one another. Nature has been sending its messages louder each day and it is no longer possible to ignore them. It is time for leaders in the Americas to step up and take practical actions to mitigate temperature rises and pollution, adapt to changes, mobilise finances, and collaborate with the rest of the world, as per COP26 goals.

